# The Rubber Stopper: A Simple and Inexpensive Technique to Prevent Pin Tract Infection following Kirschner Wiring of Supracondylar Fractures of Humerus in Children

**DOI:** 10.5704/MOJ.1507.006

**Published:** 2015-07

**Authors:** JE Santy, J Kamal, AH Abdul-Rashid, S Ibrahim

**Affiliations:** * Rumah Sakit Bedah Surabaya, Surabaya, Indonesia; ** Department of Orthopaedics and Traumatology, Universiti Kebangsaan Malaysia, Kuala Lumpur, Malaysia

**Keywords:** Kirschner wires, pin tract infection, supracondylar humerus fracture

## Abstract

Percutaneous pinning after closed reduction is commonly used to treat supracondylar fractures of the humerus in children. Minor pin tract infections frequently occur. The aim of this study was to prevent pin tract infections using a rubber stopper to reduce irritation of the skin against the Kirschner (K) wire following percutaneous pinning. Between July 2011 and June 2012, seventeen children with closed supracondylar fracture of the humerus of Gartland types 2 and 3 were treated with this technique. All patients were treated with closed reduction and percutaneous pinning and followed up prospectively. Only one patient, who was a hyperactive child, developed pin tract infection due to softening of the plaster slab. We found using the rubber stopper to be a simple and inexpensive method to reduce pin tract infections following percutaneous pinning.

## Introduction

**Fig. 1:**
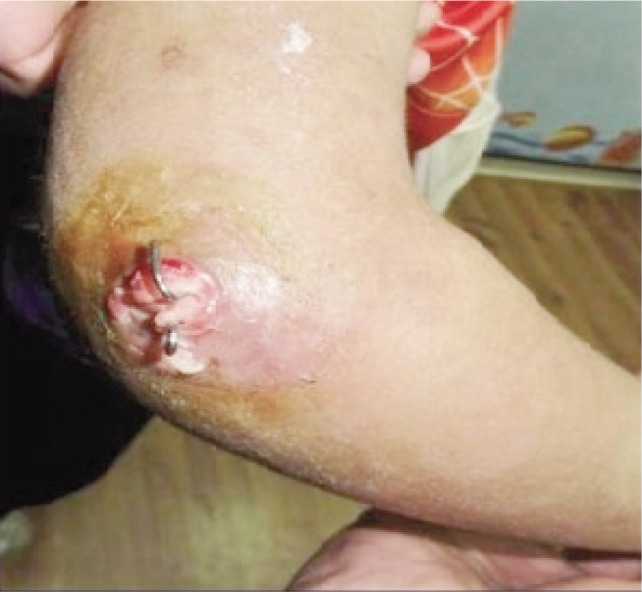
Pin tract infection in percutaneous K-wiring.

Metal wires, commonly known as Kirschner (K) wires, are used as external fixation devices in the management of orthopaedic fractures^1^. K- wires are extremely useful for fracture fixation in the paediatric population. These wires protrude through the skin and are therefore described as percutaneous. Pin tract infections frequently occur (Figure 1). The wound around the K-wire may be an entry point for microbes which result in a pin tract infection^2^.

Pin tract infection is a common and well documented complication which is treated with antibiotics and removal of pin^3^. If neglected, it leads to serious complications including osteomyelitis, septic arthritis, early physeal fusion, flexor sheath infection and toxic shock syndrome^3,4^. Pin tract infections may need to be treated with antibiotics, lead to early pin loosening and cause deep infection requiring surgical treatment^3^.

Pin tract infections occur following excessive motion of the skin around the pin or motion of the pin itself. This leads to irritation of the skin by the K-wire with movements of the elbow as a plaster slab does not provide rigid immobilization^5^.

The aim of this study was to prevent pin tract infections by using a rubber stopper to reduce movement of the skin against the K-wire following percutaneous pinning in supracondylar fractures of the humerus in children.

## Materials and Methods

Between July 2011 and June 2012, 17 children admitted to Hospital Universiti Kebangsaan Malaysia with closed supracondylar humeral fractures of Gartland types 2 and 3 were treated using this technique. There were 14 boys and three girls ranging in age from six to 12 years (average 8.7 years) and all were followed up prospectively.

**Fig. 2a, 2b and 2c:**
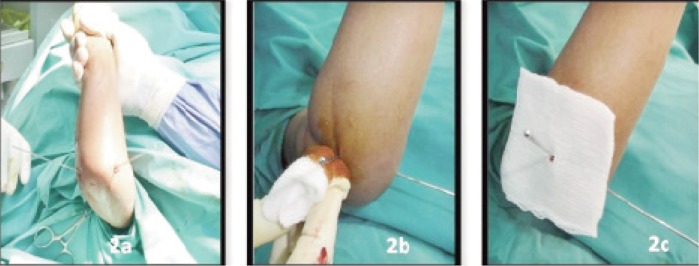
After percutaneous K- wiring, the pin site is dabbed with povidone iodine solution, and gauze dressing to prevent direct skin contact with the rubber stopper.

All the fractures were treated by closed reduction with percutaneous K-wire fixation. The procedure was performed by an orthopaedic trainee within 24 hours of admission. Closed manipulative reduction under general anaesthesia was performed and the reduction confirmed with the image intensifier. The fracture was fixed with two crossed K-wires ranging from one to 1.6 mm diameter. The medial K-wire was inserted with a mini-open technique to prevent injury to the ulnar nerve (Figure 2a).

After percutaneous K-wire insertion, the pin site was dabbed with povidone iodine solution (Figure 2b). A key hole was made in a piece of sterile gauze which was then inserted through the K-wire (Figure 2c). The rubber stoppers were obtained from empty intravenous solution bottles (Figure 3a) and sterilized. A small hole was made using another K-wire in the center of the sterilized rubber stopper. The rubber stopper was then inserted through the percutaneous K-wire and pressed snugly against the skin to ensure uniform pressure (Figure 3b).

**Fig. 3a:**
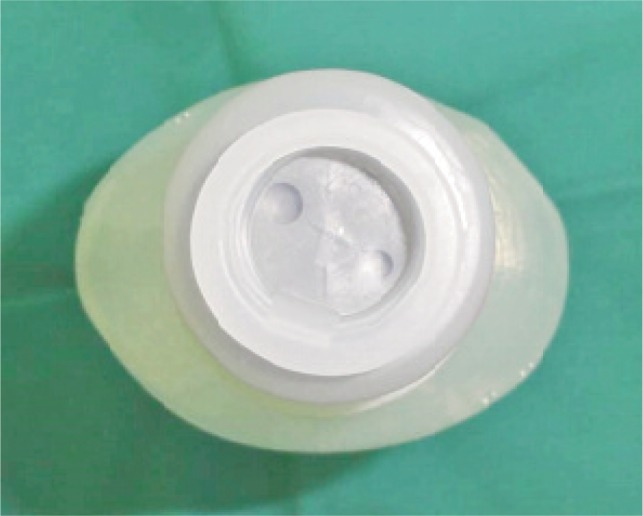
Rubber stopper from an intravenous solution bottle.

**Fig. 3b:**
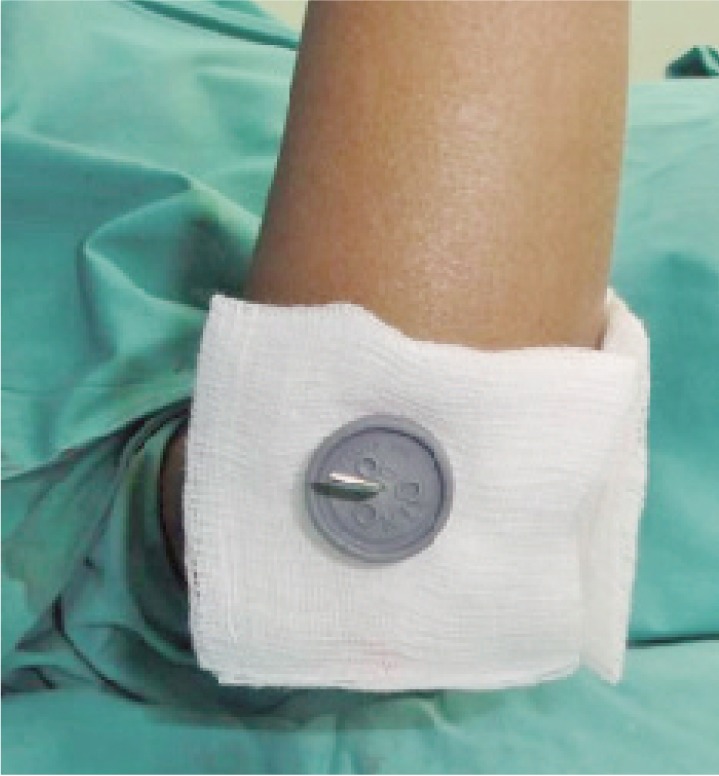
The piece of gauze prevents the rubber stopper from direct skin contact.

This - would reduce movements of the skin against the K-wire. The gauze prevented direct skin contact of the rubber stopper which may cause an allergic reaction in sensitive skin.

Pins were left protruding through the skin and the ends of the K-wire were bent to prevent migration before applying a dorsal plaster slab. The patients were discharged on the second postoperative day.

All the patients were reviewed in the clinic after one week to ensure that the plaster slab had not softened or loosened. The pin-sites were not routinely inspected at the one week follow-up. The pin-sites dressing and plaster slab were changed only if necessary. The plaster slab and K-wire were removed three weeks after surgery. The assessment consisted of a clinical examination and scoring of pin tracts using the Checketts-Otterburn’s grading system for level of pin-site infection^6^.

Pin tract infection was diagnosed when there was observation of seropurulent discharge or erythema around the pin site, with or without bacteriological evidence of infection or if the pin tract score was grade two or higher^6^.

## Results

Seventeen patients were recruited in this study. All patients were successfully treated with closed reduction and percutaneous cross-pinning.

One patient, a hyperactive child, developed pin tract infection after one week. The plaster slab had softened and one K-wire had dislodged. He recovered with oral antibiotic and pin site care.

**Fig. 4:**
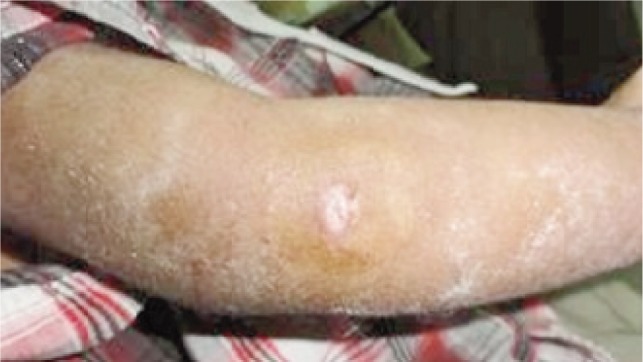
No pin tract infection after removal of rubber stopper and gauze at 3 week.

The other 16 patients did not develop pin-site infections (Figure 4). None of these patients needed the pin-site dressings to be changed after the first week.

## Discussion

The recommended treatment for supracondylar fractures of the humerus of Gartland types 2 and 3 is closed reduction and percutaneous pinning^7^. Pin tract infections are common.

The exposed ends of the percutaneous K-wires are vulnerable to pin tract infections. Although pin tract infection is often not considered to be a serious complication in the short term, it has the potential to decrease the stability of the bone–pin interface, resulting in pin loosening, osteomyelitis and a poor functional outcome^8^.

In previously published studies, the pin tract infection rates were reported to be between 7.5% to 19.7% after percutaneous K-wiring of supracondylar humeral fractures in children^2,9,10^. The only patient in the present study who developed pin tract infection was a six-year-old hyperactive boy. The plaster slab had softened resulting in increased skin irritation. This was a grade 2 infection based on CheckettsOtterburn classification. The infection healed with local wound care and oral antibiotic therapy.

A number of strategies have been employed to reduce pin site sepsis. These included pin site cleaning, release of tethered skin, coating pins with antibacterial substances, prophylactic application of topical antibiotics, pin insertion techniques and a wide variety of protocols for pin site care^3^. However, there was no evidence to suggest that these practices reduced the infection rate and there was little published data on the actual infection rates with K-wire.

In our study, the dressings were not changed after the first postoperative week on the premise that the less one disturbed the wound site the less was the risk of contamination^11^. The use of the rubber stopper to hold the dressing and restrict movement of the K-wire against the skin was adapted from the technique with the Ilizarov external fixator pin-sites^12^.

## Conclusion

We found the rubber stopper to be a simple and inexpensive method to prevent pin-tract infections. We recommend using this technique not only in the elbow but in other areas where percutaneous K-wiring is used.
